# Health-related behavioral changes and incidence of chronic kidney disease: The Japan Specific Health Checkups (J-SHC) Study

**DOI:** 10.1038/s41598-022-20807-2

**Published:** 2022-09-29

**Authors:** Hiroshi Kimura, Koichi Asahi, Kenichi Tanaka, Kunitoshi Iseki, Toshiki Moriyama, Kunihiro Yamagata, Kazuhiko Tsuruya, Shouichi Fujimoto, Ichiei Narita, Tsuneo Konta, Masahide Kondo, Masato Kasahara, Yugo Shibagaki, Tsuyoshi Watanabe, Junichiro J. Kazama

**Affiliations:** 1 Department of Nephrology and Hypertension, Fukushima Medical University, 1 Hikariga-oka, Fukushima City, 960-1295 Japan; 2 Division of Nephrology and Hypertension, Iwate Medical University, Yahaba, Japan; 3 Steering Committee of The Japan Specific Health Checkups (J-SHC) Study Group, Fukushima, Japan

**Keywords:** Outcomes research, Nephrology, Risk factors

## Abstract

The transtheoretical model (TTM) is a commonly used model of health-related behavioral change. However, the practical effect of using this model for chronic kidney disease (CKD) self-management remains unclear. This study aimed to investigate the association between stages of change for lifestyle behavior and the incidence of CKD in the general Japanese population. A retrospective cohort study was conducted among 178,780 non-CKD participants aged 40–74 years who underwent annual health check-ups for two consecutive years between 2008 and 2009. Health behavior change was determined using questionnaires based on the TTM, which consists of five stages of change (precontemplation, contemplation, preparation, action, and maintenance). The exposure of interest was the change in stages between two years. Participants were categorized into 3 groups ‘improved’, ‘unchanged’, or ‘deteriorated’. The association between the change in stages and the incidence of CKD was examined using logistic regression analysis. After one year of follow-up, 20.0% of participants developed CKD. Participants in the deteriorated group showed a significantly higher risk of CKD incidence than in the improved group. Promoting the stage of change for healthy lifestyle behaviors evaluated by the TTM was associated with a risk reduction for the incidence of CKD.

## Introduction

The incidence and prevalence of chronic kidney disease (CKD) continue to increase worldwide^1^. CKD is a progressive disease that leads to end-stage kidney disease, and is associated with an increased risk of cardiovascular disease (CVD) and mortality^2^. Because nearly one in eight adults have CKD in Japan^3^, the Japanese government assigned CKD as a national target disease for strategic medical research in 2007^4,5^. Recent investigations of research priority settings for kidney disease identified the need to establish optimal strategies to enable patients to manage their CKD and related comorbidities, and highlighted self-management as a top priority to prevent progression of CKD^6,7^. Nevertheless, effective self-management strategies for patients with CKD remain unclear.

Behavioral change toward a healthy lifestyle is a key component of self-management of CKD. The transtheoretical model (TTM), which includes five stages of change (precontemplation, contemplation, preparation, action, and maintenance), is a behavioral change model that can guide health care providers to understand individuals’ motivation toward establishing and maintaining health-related behavioral changes^8^. The TTM has been useful in several studies on smoking cessation^9,10^, improving eating habits, and promoting physical activity^11,12^. Furthermore, a recent systematic review suggested that the TTM can be applied to prevent chronic diseases^13^. However, a limited number of studies have investigated the practical use of the TTM in the field of CKD.

The purpose of this study was to examine the association between change in stage of the TTM and the incidence of CKD in a Japanese nationwide population that underwent health check-ups over two consecutive years between 2008 and 2009.

## Results

### Participants characteristics

**Figure 1:**
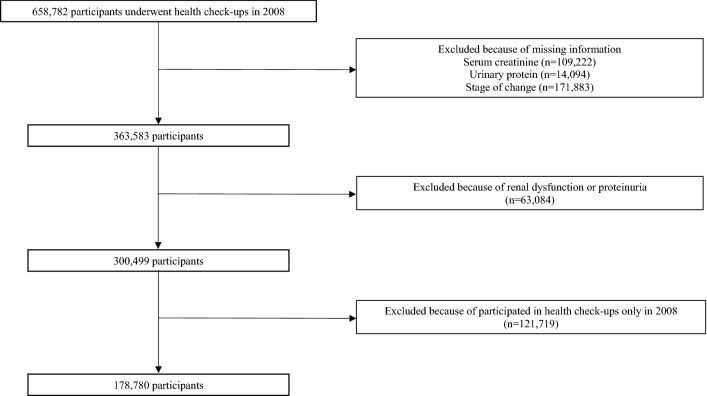
Flow chart showing the selection of the study participants.

Of the 658,782 individuals who participated in health check-ups in 2008, we excluded those who had missing information on serum creatinine (n = 109,222), urinalysis (n = 14,094), and the stage of change for health behavior (n = 171,883). Then, we excluded participants with CKD at baseline (i.e., 2008) (n = 63,084). Finally, we excluded those who had not participated in health check-ups in 2009 (n = 121,719). Thus, a total of 178,780 were included in this study (Fig. 1). Compared to the 121,719 excluded participants without CKD who underwent a health check-up only in 2008, the included participants were older and less likely to be smokers (Supplemental Table 1).

Among the 178,780 included participants, 54,870 (30.7%), 55,034 (30.8%), 20,630 (11.5%), 14,059 (7.9%), and 34,187 (19.1%) were in the precontemplation, contemplation, preparation, action, and maintenance stages of change, respectively. Participants in the precontemplation and maintenance stages were older, more likely to be male, and had lower BMIs, lower waist circumferences, and lower low-density lipoprotein cholesterol levels than those in other stages. The frequency of smoking decreased in the more active stages of change, and daily drinkers showed a similar trend. The prevalence of hypertension and dyslipidemia increased between the precontemplation and action stages but decreased in the maintenance stage, while the prevalence of diabetes increased between the precontemplation and maintenance stages. After one year of follow-up, more than half of the participants in the precontemplation and maintenance stages had the same stages as those at baseline. However, participants in the contemplation, preparation, and action stages changed to different stages; in particular, participants in the preparation stage tended to change to contemplation, and participants in action stage tended to move into the maintenance stage (Table 1).

**Table 1:** Clinical characteristics of 178,780 participants according to the stage of healthy lifestyle change.

	Total	Precontemplation	Contemplation	Preparation	Action	Maintenance	
	n = 178,870	n = 54,870	n = 55,034	n = 20,630	n = 14,059	n = 34,187	P trend
**Baseline (2008)**							
Age, years	66 (61–70)	67 (62–70)	65 (59–69)	65 (59–69)	65 (60–69)	67 (63–70)	0.002
Men, %	37	44	34	32	33	36	< 0.001
Body mass index, kg/m^2^	22.5 (20.6–24.6)	22.0 (20.2–24.0)	22.9 (20.9–25.1)	23.0 (21.0–25.2)	23.0 (21.1–25.0)	22.3 (20.6–24.2)	< 0.001
Waist, cm	83.0 (77.0–88.6)	81.5 (75.5–87.0)	84.0 (78.0–90.0)	84.0 (78.0–90.0)	84.0 (78.0–89.7)	82.2 (77.0–88.0)	< 0.001
Systolic BP, mmHg	128 (118–140)	128 (118–140)	128 (118–140)	128 (118–140)	128 (118–140)	129 (118–140)	0.002
Diastolic BP, mmHg	76 (70–82)	76 (70–82)	76 (70–82)	76 (70–82)	76 (70–82)	76 (70–82)	0.47
Fasting plasma glucose, mg/dL	93 (87–101)	92 (86–99)	93 (87–100)	93 (87–101)	93 (87–102)	93 (87–102)	< 0.001
Hemoglobin A_1c_, %	5.6 (5.4–5.9)	5.6 (5.4–5.8)	5.6 (5.4–5.9)	5.6 (5.4–5.9)	5.7 (5.4–5.9)	5.7 (5.4–5.9)	< 0.001
Triglyceride, mg/dL	98 (71–137)	94 (69–132)	101 (73–144)	101 (73–144)	100 (74–139)	95 (70–132)	0.817
HDL cholesterol, mg/dL	62 (52–73)	62 (52–74)	61 (51–72)	61 (51–72)	61 (51–72)	63 (53–74)	0.002
LDL cholesterol, mg/dL	124 (105–144)	121 (103–141)	126 (107–146)	127 (108–148)	126 (107–146)	124 (105–144)	< 0.001
Creatinine, mg/dL	0.64 (0.60–0.70)	0.70 (0.60–0.80)	0.60 (0.60–0.70)	0.60 (0.60–0.70)	0.61 (0.60–0.70)	0.68 (0.60–0.71)	< 0.001
eGFR, ml/min/1.73m^2^	75.7 (68.6–88.5)	75.3 (69.4–88.5)	76.0 (69.9–89.7)	75.7 (68.5–89.0)	75.7 (67.8–87.1)	75.0 (66.4–86.1)	< 0.001
Uric acid, mg/dL	4.9 (4.1–5.7)	4.9 (4.1–5.8)	4.8 (4.1–5.7)	4.9 (4.1–5.7)	4.9 (4.1–5.8)	4.9 (4.1–5.7)	0.75
Hemoglobin, g/dL	13.5 (12.6–14.4)	13.4 (12.5–14.4)	13.4 (12.6–14.4)	13.5 (12.6–14.4)	13.5 (12.7–14.4)	13.5 (12.7–14.4)	< 0.001
Smoke, %	13	16	15	13	10	8	< 0.001
Daily drinker, %	46	50	45	44	44	44	< 0.001
Hypertension, %	47	45	46	48	49	48	< 0.001
Diabetes, %	9	6	9	9	11	12	< 0.001
Dyslipidemia, %	43	37	45	48	48	46	< 0.001
Cardiovascular disease, %	9	6	9	9	11	10	< 0.001
Antihypertensive drug, %	28	37	28	48	31	29	< 0.001
Antidiabetic drug, %	4	8	4	9	6	7	< 0.001
Antilipidemic drug, %	16	26	16	28	20	20	< 0.001
**Follow-up (2009)**							
Stage of changes, %							
Precontemplation	31.3	61.0	19.2	15.5	15.9	19.2	
Contemplation	28.7	17.6	47.6	34.1	23.6	14.8	
Preparation	11.8	6.2	14.4	24.6	14.5	7.7	
Action	7.7	4.1	7.4	11.1	15.9	8.3	
Maintenance	20.5	11.1	11.3	14.7	30	50.1	
Incidence of CKD, %	20	19.8	19.0	20.6	21.5	20.8	< 0.001
Incidence of proteinuria, %	2.8	2.8	2.9	2.9	2.8	2.5	0.001

Values are expressed as medians (interquartile range), or percentage as appropriate.

*BP* blood pressure, *HDL* high-density lipoprotein, *LDL* low-density lipoprotein, *eGFR* estimated glomerular function rate, *CKD* chronic kidney disease.

When we divided participants into three groups as their change in the stage of lifestyle behaviors between two years, 49,202 (27.5%), 84,121 (47.1%), and 45,457 (25.4%) were in the improved, unchanged, and deteriorated group, respectively. Participants in the unchanged group had lower BMI and waist circumferences, and tended to show lower proportion of comorbidities than other groups (Table 2).

**Table 2:** Clinical characteristics of 178,780 participants according to stage of change for lifestyle behaviors between two years.

	Improved	Unchanged	Deteriorated	P trend
	n = 49,202	n = 84,121	n = 45,457	
Age, years	66 (60–69)	66 (60–70)	66 (61–70)	< 0.001
Men, %	36	39	35	0.034
Body mass index, kg/m^2^	22.8 (20.8–24.9)	22.3 (20.5–24.3)	22.7 (20.8–24.7)	< 0.001
Waist, cm	83.7 (77.5–89.3)	82.4 (76.2–88.0)	83.0 (77.0–89.0)	< 0.001
Systolic BP, mmHg	129 (118–140)	128 (118–139)	128 (118–140)	0.001
Diastolic BP, mmHg	76 (70–82)	76 (70–82)	76 (70–82)	< 0.001
Fasting plasma glucose, mg/dL	93 (87–101)	93 (87–100)	93 (87–101)	0.018
Hemoglobin A_1c_, %	5.6 (5.4–5.9)	5.6 (5.4–5.9)	5.6 (5.4–5.9)	< 0.001
Triglyceride, mg/dL	99 (73–141)	96 (70–135)	98 (72–137)	< 0.001
HDL cholesterol, mg/dL	61 (51–72)	62 (52–74)	61 (52–73)	0.001
LDL cholesterol, mg/dL	126 (107–147)	123 (104–143)	124 (106–144)	< 0.001
Creatinine, mg/dL	0.64 (0.60–0.70)	0.66 (0.60–0.71)	0.62 (0.60–0.70)	0.003
eGFR, ml/min/1.73 m^2^	75.4 (67.9–88.1)	75.7 (69.3–88.9)	75.4 (67.9–88.5)	0.974
Uric acid, mg/dL	4.9 (4.1–5.7)	4.9 (4.1–5.7)	4.8 (4.1–5.7)	0.202
Hemoglobin, g/dL	13.5 (12.6–14.4)	13.5 (12.6–14.4)	13.4 (12.6–14.4)	0.166
Smoke, %	13	14	12	0.051
Daily drinker, %	46	47	45	0.060
Hypertension, %	47	46	48	0.053
Diabetes, %	10	8	9	0.138
Dyslipidemia, %	46	42	45	< 0.001
Cardiovascular disease, %	10	9	9	< 0.001
Antihypertensive drug, %	46	27	45	< 0.001
Antidiabetic drug, %	9	4	10	< 0.001
Antilipidemic drug, %	27	16	30	< 0.001

Values are expressed as medians (interquartile range), or percentage as appropriate.

*BP* blood pressure, *HDL* high-density lipoprotein, *LDL* low-density lipoprotein, *eGFR* estimated glomerular function rate, *CKD* chronic kidney disease.

### Incidence of CKD and proteinuria with stage of change

Of the 178,780 participants, 35,734 (20.0%) developed CKD in 2009. Participants in the deteriorated group showed significantly higher odds for the incidence of CKD. In an unadjusted model, unchanged and deteriorated groups had graded associations with the incidence of CKD using the improved group as reference. These associations remained significant after additional adjustments in Model 1 and Model 2. The adjusted odds ratios for incidence of CKD in Model 2 were 1.02 (95% confidence interval: 0.99–1.05) and 1.07 (95% confidence interval: 1.04–1.11) (Table 3).

**Table 3:** Logistic regression models for the association between stage of change and incidence of CKD, proteinuria.

	Number of events/total (%)	Unadjusted odds ratios (95% CI)	Adjusted odds ratios (95% CI)	
			Model 1	Model 2
**Incidence of CKD**				
Improved	9,620/49,202 (19.6)	Reference		
Unchanged	16,675/84,121 (19.8)	1.02 (0.99–1.05)	1.02 (0.99–1.05)	1.02 (0.99–1.05)
Deteriorated	9,439/45,457 (20.8)	1.08 (1.04–1.11)	1.08 (1.04–1.11)	1.07 (1.04–1.11)
**Incidence of Proteinuria**				
Improved	1,328/49,202 (2.7)	Reference		
Unchanged	2,325/84,121 (2.8)	1.02 (0.96–1.10)	1.04 (0.97–1.11)	1.05 (0.98–1.12)
Deteriorated	1,333/45,457 (2.9)	1.09 (1.01–1.18)	1.10 (1.02–1.19)	1.10 (1.01–1.18)

*CKD* chronic kidney disease.

The incidence of proteinuria was observed in 4,986 (2.8%) participants in 2009. Similar to the incidence of CKD, graded associations were observed in the unchanged and deteriorated group when we used the improved group as a reference. The adjusted odds ratios for incidence of proteinuria in Model 2 were 1.05 (95% confidence interval: 0.98–1.12) and 1.10 (95% confidence interval: 1.01–1.18), respectively.

## Discussion

This nationwide retrospective cohort study revealed that about 30% of health check-up participants in Japan without CKD were not interested in changing toward a healthy lifestyle. In addition, another 30% had an interest but had not changed their lifestyle behaviors. Although more than half of participants in the precontemplation and maintenance stages did not change or maintained their stages after one year of follow-up, those in the middle stages (i.e., contemplation, preparation, action stage) shifted to various stages, suggesting that deterioration and improvement of stages of change were common in the general population without CKD. Furthermore, participants in the deteriorated group showed significantly higher odds for the incidence of CKD and proteinuria. Our current findings support the view that a better stage of change in lifestyle behaviors might be important to prevent the development of CKD and proteinuria.

Previous observational studies have shown that several unhealthy lifestyle factors, such as obesity, weight gain after maturity, poor diet quality, smoking, and heavy alcohol consumption, were independently associated with CKD and proteinuria^14–17^. It has been reported that a combination of unhealthy lifestyles or not changing unhealthy lifestyle behaviors was associated with an increased risk of incidence of CKD and proteinuria^18–21^. Moreover, the accumulation of healthy lifestyle behaviors, especially those related to habitual moderate exercise and no bedtime snacking, was suggested to reduce the risk of CKD^22^. The present study, with a sufficient number of participants, provided evidence that deterioration of stage of change for a healthy lifestyle between a year was associated with the development of CKD and proteinuria.

Although health behavior changes toward a healthy lifestyle are a key component of prevention for CKD, the optimal method for promoting changes in health behaviors for CKD self-management has not been clarified. The TTM is one of the most commonly used methods in behavioral change modeling and has been used for prevention interventions for chronic diseases like diabetes, hypertension, and cancer^13^. With respect to CKD, a randomized controlled trial of 160 patients with early-stage CKD showed that targeted interventions matched to the TTM stages of change promoted adherence to proper diet, exercise behavior, and positive lifestyle modifications^23^. The findings of our study support interventions for healthy lifestyle behaviors matched to the TTM might reduce the incidence of CKD and proteinuria.

We observed that 20% of participants developed CKD in this study. Surprisingly, participants in the promoted stages (i.e., preparation, action, maintain stage) at baseline showed a higher incidence rate of CKD. We have speculated that participants with promoted stages had a higher prevalence of comorbidities, resulting in a higher risk of CKD development. Approximately 30% of participants were in the precontemplation stage, and this group had lower BMIs as well as better glycemic and lipid control than those in the contemplation, preparation, and action stages. From these observations, we speculated that many participants in the precontemplation stage were healthier with no health problems. Therefore, they were not interested in making changes to healthier lifestyles. On the other hand, participants in the maintenance stage had similar BMI and lipid control to those in the precontemplation stage. Although they had higher rates of prevalence of diabetes and dyslipidemia, they showed significantly lower risk of proteinuria relative to those in other groups. These findings further support the argument that maintaining a healthy lifestyle reduces the risk of proteinuria^18^.

A major strength of this study was its large-scale longitudinal nature, with participants from throughout Japan. However, several limitations of this study should be mentioned. First, the stages of change were determined based on information obtained through a self-report questionnaire, thus their responses may not be accurate. Second, we were not able to assess which lifestyle modification affected to reduce the risk of the incidence of CKD. Healthy lifestyle behaviors, such as exercising and healthy eating habits, might reduce the risk of CKD. However, we could not obtain this information in this study. Third, there may have been selection bias because the included subjects received annual health check-ups for two consecutive years, and thus these subjects may have a higher interest in their health. In fact, excluded subjects who did not have CKD and underwent health check-ups only in 2008 had a higher proportion of smokers. Therefore, included participants might be a relatively health-conscious, and the stages of change for lifestyle behaviors may be underestimated in this study population. Fourth, we could not assess the kind of antihypertensive medication, such as angiotensin receptor blockers or angiotensin-converting enzyme inhibitors, which may affect both renal function and proteinuria, due to not including in this database. Finally, unmeasured or residual confounding factors may still exist even though we adjusted for potential confounding factors.

In conclusion, the improved stages of change for healthy lifestyle behaviors were associated with reduced risk of incidence of CKD and proteinuria. Our results indicate that appropriate intervention strategies for promoting healthy lifestyle behaviors could substantially reduce the incidence of CKD. Further studies are needed to investigate whether an improved lifestyle minimizes the incidence of cardiovascular disease or mortality.

## Methods

### Study population

This retrospective cohort study used data from the Specific Health Check and Guidance, which was initiated by the Japanese government to promote the early diagnosis of metabolic syndrome and establish prompt intervention in 2008. Clinical details of this cohort have been described previously^24–26^. We obtained data from 15 prefectures (Hokkaido, Miyagi, Fukushima, Niigata, Tokyo, Kanagawa, Ishikawa, Nagano, Osaka, Tokushima, Fukuoka, Saga, Nagasaki, Miyazaki, and Kumamoto) whose local governments agreed to participate. We used participants without CKD, aged 40–74 who received their periodic health check–up in both 2008 and 2009.

The study was performed in accordance with the Declaration of Helsinki and Ethical Guidelines for Epidemiological Studies published by the Ministry of Education, Science and Culture and the Ministry of Health, Labour and Welfare of Japan. The requirement for informed consent was waived because the data were anonymous. The study protocol was granted ethical approval by Fukushima Medical University (IRB Approval Number #1485, #2771).

### Measurement and definition

All participants answered a self-administered questionnaire that covers medical history, smoking habits, alcohol intake, exercise habits, and eating patterns. The stage of change for lifestyle behaviors was assessed by a questionnaire consisting of a 5-item algorithm, based on the TTM. The first item asked if respondents intended to change their lifestyle in the next six months; patients who responded “No” were considered in the pre-contemplation stage, while those who responded “Yes” were in the contemplation stage. Respondents who answered that they were going to change their lifestyle in the next few months were in the preparation stage. Respondents who had already started appropriate actions, such as reducing dietary fat and starting exercise within the past six months, were in the action stage. Respondents who had been taking action for more than six months were in the maintenance stage.

Participants were considered to have a history of CVD if they responded “yes” to either of the questions, “Have you ever been told that you have had a stroke or have you received treatment for stroke?” or “Have you ever been told that you have heart disease or have you received treatment for heart disease?”.

The physicians involved in this study performed a physical examination of each participant and rechecked their medical history to verify the precision of the information. Trained staff measured participants’ height, weight, waist circumference, and blood pressure. The body mass index (BMI) was calculated as the ratio of body weight (kg) to height squared (m^2^). Blood pressure was measured using a standard sphygmomanometer or an automated device while in a seated position after the subject had rested for 5 min. Blood and urine samples were collected after an overnight fast and assayed within 24 h.

Hypertension was defined as the use of antihypertensive drugs, a systolic blood pressure ≥ 140 mmHg and/or a diastolic blood pressure ≥ 90 mmHg, or both. Diabetes was defined in accordance with American Diabetes Association Guidelines, and was identified by a fasting plasma glucose concentration ≥ 126 mg/dL, a glycated hemoglobin (HbA1c) value ≥ 6.5%, or the use of an antidiabetic drug. The HbA1c value was estimated using the National Glycohemoglobin Standardization Program equivalent value calculated using the following equation: HbA1c (%) = HbA1c (Japan Diabetes Society) + 0.4%^27^. Dyslipidemia was defined as the use of antilipidemic drugs, a low-density lipoprotein cholesterol concentration ≥ 140 mg/dL, or both^28^. We defined CKD as estimated glomerular filtration rate (eGFR) < 60 mL/min/1.73 m^2^, or proteinuria on urinalysis, or both. The eGFR was calculated using the formula for Japanese^29^. The results of urinalysis were recorded as (−), ( ±), (1 +), (2 +), and (3 +), and the presence of proteinuria was defined as a dipstick urinalysis score ≥ (1 +).

### Exposure and outcomes

The exposure of interest for this study was stage of change for lifestyle behaviors between two years. Participants were categorized into three groups ‘improved’ (changed from worse to the better stage), ‘unchanged’ (the same stage), or ‘deteriorated’ (changed from better to the worse stage). The primary outcome was the incidence of CKD, and the secondary outcome was the incidence of proteinuria.

### Statistical analysis

All variables were reported as medians with interquartile ranges, or frequency (percent) as appropriate. Differences in baseline characteristics between included versus excluded participants were compared by standardized differences because of the large sample size of this study^30,31^. Nonparametric trend tests (Jonckheere–Terpstra trend test or a Cochran–Armitage trend test^32–35^) evaluated differences in baseline characteristics across the stage of change and change in stage categories. Associations between the incidence of CKD and the change in stage categories were examined by logistic regression analysis. We used hierarchical adjustment with two models as follows: (1) Model 1, which adjusted for age, sex, BMI, and serum creatinine; (2) Model 2, which included the above variables plus smoking status, drinking status, and comorbidities (hypertension, diabetes, and dyslipidemia), and history of CVD. The frequency of missing data was low (< 1% for most variables, except for hypertension [12%] and CVD [9%]), and the multiple imputation method with 20 datasets was used in all regression analyses. All analyses were conducted using STATA MP, version 15.1 (Stata Corp, College Station, TX).

## Supplementary Information

Supplementary Table 1.

## Data Availability

The datasets generated and/or analyzed during the current study are available from the corresponding author on reasonable request.
