# Supplementary material for: Health-related behavioral changes and incidence of chronic kidney disease: The Japan Specific Health Checkups (J-SHC) Study
Source: Sci Rep. 2022 Sep 29;12:16319. doi: 10.1038/s41598-022-20807-2 (PMC9522825; doi:10.1038/s41598-022-20807-2)
Supplement: Supplementary file 1 — Supplementary Table 1. [file 41598_2022_20807_MOESM1_ESM.pdf]

Supplemental Table 1. Clinical characteristics of included and excluded participants.

	Included<br>n=178,780	Excluded<br>n=121,719	StdDiff.
Age, year	66 (61-70)	65 (57-70)	22.1%
Men, %	37	39	3.4%
Body mass index, kg/m^2	22.5 (20.6-24.6)	22.6 (20.6-24.9)	4.4%
Waist, cm	83.0 (77.0-88.6)	83.0 (77.0-89.0)	3.2%
Systolic BP, mmHg	128 (118-140)	128 (118-140)	2.0%
Diastolic BP, mmHg	76 (70-82)	76 (70-83)	6.0%
Fasting plasma glucose, mg/dL	93 (87-101)	93 (87-101)	6.2%
Hemoglobin A_{1c}, %	5.6 (5.4-5.9)	5.6 (5.4-5.9)	0.3%
Triglyceride, mg/dL	98 (71-137)	99 (72-142)	5.4%
HDL cholesterol, mg/dL	62 (52-73)	61 (51-73)	2.6%
LDL cholesterol, mg/dL	124 (105-144)	124 (104-145)	0.8%
Creatinine, mg/dL	0.64 (0.60-0.70)	0.67 (0.60-0.71)	1.9%
eGFR, $ml/min/1.73m^2$	75.7 (68.6-88.5)	76.3 (70.1-89.4)	7.2%
Uric acid, mg/dL	4.9 (4.1-5.7)	4.9 (4.1-5.8)	3.3%
Hemoglobin, g/dL	13.5 (12.6-14.4)	13.5 (12.6-14.5)	6.2%
Smoke, %	13	19	14.7%
Daily drinker, %	46	49	6.1%
Hypertension, %	47	49	4.0%
Diabetes, %	9	11	7.0%
Dyslipidemia, %	43	43	0.9%
Cardiovascular disease, %	9	11	0.1%
Antihypertensive drug, %	28	43	3.0%
Antidiabetic drug, %	4	9	3.2%
Antilipidemic drug, %	16	27	5.8%

Note: Values are expressed as medians (interquartile range), or percentage as appropriate. Standardized difference of $\geq$10% was defined as meaningful imbalance against 121,719 excluded participants. Abbreviations: BP, blood pressure; HDL, high-density lipoprotein; LDL, low-density lipoprotein; eGFR, estimated glomerular function rate; StdDiff, standardized difference.
